# Patient-specific PEEK implants for immediate restoration of temporal fossa after maxillary reconstruction with temporalis muscle flap

**DOI:** 10.1186/s40902-022-00348-4

**Published:** 2022-05-07

**Authors:** Sherif Ali, Omniya Abdel Aziz, Mamdouh Ahmed

**Affiliations:** 1 Oral and Maxillofacial Surgery Department, Faculty of Dentistry, Cairo University, Cairo, Egypt; 2 Oral and Maxillofacial Surgery Department, Nasser Institute for Treatment and Research, Cairo, Egypt

**Keywords:** Maxillofacial reconstruction, Temporalis flap, Temporal hollowing, Computer-assisted surgery, Patient-specific implants, Rapid prototyping

## Abstract

**Background:**

Temporal hollowing is a common complication following the rotation of the temporalis muscle that leaves the patient with a cosmetic impairment. Several alloplastic materials have been used to reconstruct the donor site; however, these implants need meticulous adaptation to conform the periphery of the defect and restore the contour of the temporal area. The aim of this study was to assess the use of patient-specific polyetheretherketone (PEEK) temporal implants to prevent temporal hollowing following the use of full temporalis muscle flap for large maxillary defects reconstruction.

**Methods:**

This was a prospective study conducted on eight patients with major maxillary defects indicating the need of reconstruction with full temporalis muscle flap or any lesion indicating major maxillary resection and immediate reconstruction with total temporalis muscle flap. For each patient, a patient-specific PEEK implant was fabricated using virtual planning and milled from PEEK blocks. In the surgical theater, the temporalis muscle was exposed, elevated, and transferred to the maxilla. After the temporalis muscle transfer, PEEK implants were fixed in place to prevent temporal hollowing.

**Results:**

The surgical procedures were uneventful for all patients. The esthetic result was satisfactory with no post-operative complications except in one patient where seroma occurred after 2 weeks and resolved after serial aspiration.

**Conclusion:**

Patient-specific PEEK implant appears to facilitate the surgical procedures eliminate several meticulous steps that are mainly based on the surgeon’s experience.

**Trial registration:**

Clinical trials registration: NCT05240963.

## Introduction

Temporalis muscle flap has been widely used as a pedicled flap in head and neck reconstruction. Due to its versatility and reliability, it can be used in many situations to replace missing tissues such as oral defects, hard and soft palate, skull base, malar bone, mastoid cavities, and orbital defects obliteration. Moreover, temporalis muscle flap-associated complications such as flap necrosis, hematoma, seroma, and facial nerve injury are relatively uncommon. Nevertheless, temporal hollowing represents a true concern that can hinder the use of temporalis muscle flap [1, 2].

Temporal hollowing (depression) is a common complication following the rotation of the temporalis muscle that leaves the patient with a cosmetic impairment. The muscle transposition results in depression of the temporal region soft tissue contour, aggravated with the elevated appearance of the orbital rim and zygomatic arch [3]. In a trial to overcome the hollowing problem, the split temporalis muscle flap has been previously used. The muscle is split into anterior and posterior parts based on the anterior and posterior deep temporal arteries. The posterior part is then used for the defect repair, while the anterior half is left to avoid temporal hollowing. In another approach, the anterior part is transferred to the recipient site, while the remaining posterior part is transposed forward to the anterior region of the temporal fossa to mask the depression lateral to the orbital margin [4, 5]. However, this approach results in questionable esthetic outcomes, especially in bald patients. Moreover, it can be used only in small defects where a portion of the muscle is sufficient to repair the defect. While, in many cases, the use of a whole muscle is mandatory indicating the need for other solutions [6].

For several years, temporal hollowing has been camouflaged by hair styling; however, this was not satisfactory; moreover, it is not applicable in bald patients indicating the need for temporal fossa augmentation [7]. Several autogenous materials have been used to reconstruct the donor site and prevent temporal hollowing, but they showed questionable results compared with the emerging alloplastic materials [3]. Various alloplastic materials have been previously used as polymethyl methacrylate (PMMA) cement and porous high-density polyethylene (PHDPE) implants, nevertheless these implants need meticulous adaptation to conform the periphery of the defect and restore the contour of the temporal area [7–10].

The use of computer-assisted surgery (CAS) has been popular in maxillofacial surgery. Virtual planning and simulation of the surgical procedure can be executed with the aid of computer technologies; furthermore, rapid prototyping allowed the printing of physical human models derived from the radiographic images. Recently, a drastic shift occurred in CAS due to the quick evolution in planning software and rapid prototyping technology, moving from virtual planning to more patient-specific hardware as patient-specific implants [11]. The aim of this study was to assess the use of patient-specific polyetheretherketone (PEEK) temporal implants to prevent temporal hollowing following the use of full temporalis muscle flap for large maxillary defects reconstruction.

## Materials and methods

This was a prospective case series study conducted on eight consecutive patients. The patients were selected according to the following criteria: Patients with major maxillary defects indicating the need of reconstruction with full temporalis muscle flap; or any lesion indicating major maxillary resection and immediate reconstruction with total temporalis muscle flap. For all patients, immediate donor site reconstruction was performed using patient-specific PEEK temporal implants. The surgical procedures were performed in Oral and Maxillofacial Department, Faculty of Dentistry, Cairo University. This study was approved by the institution research committee and followed the Declaration of Helsinki on medical research.

**Fig. 1:**
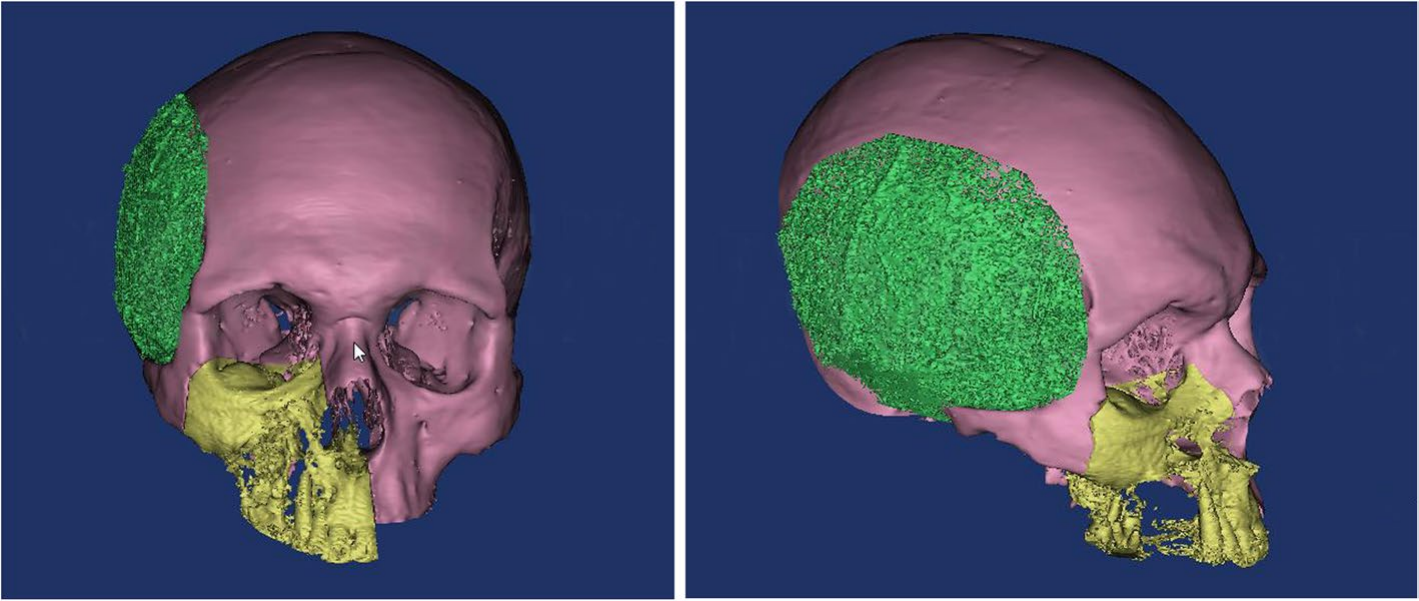
The temporalis muscle virtually segmented and separated (green)

**Fig. 2:**
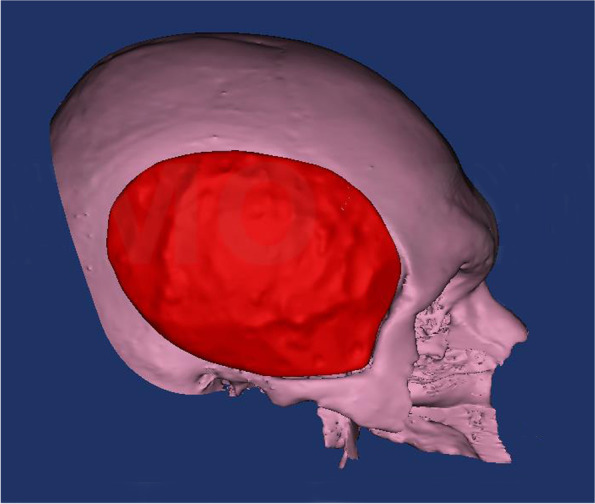
The muscle refined to create the final virtual temporal implant (red)

**Fig. 3:**
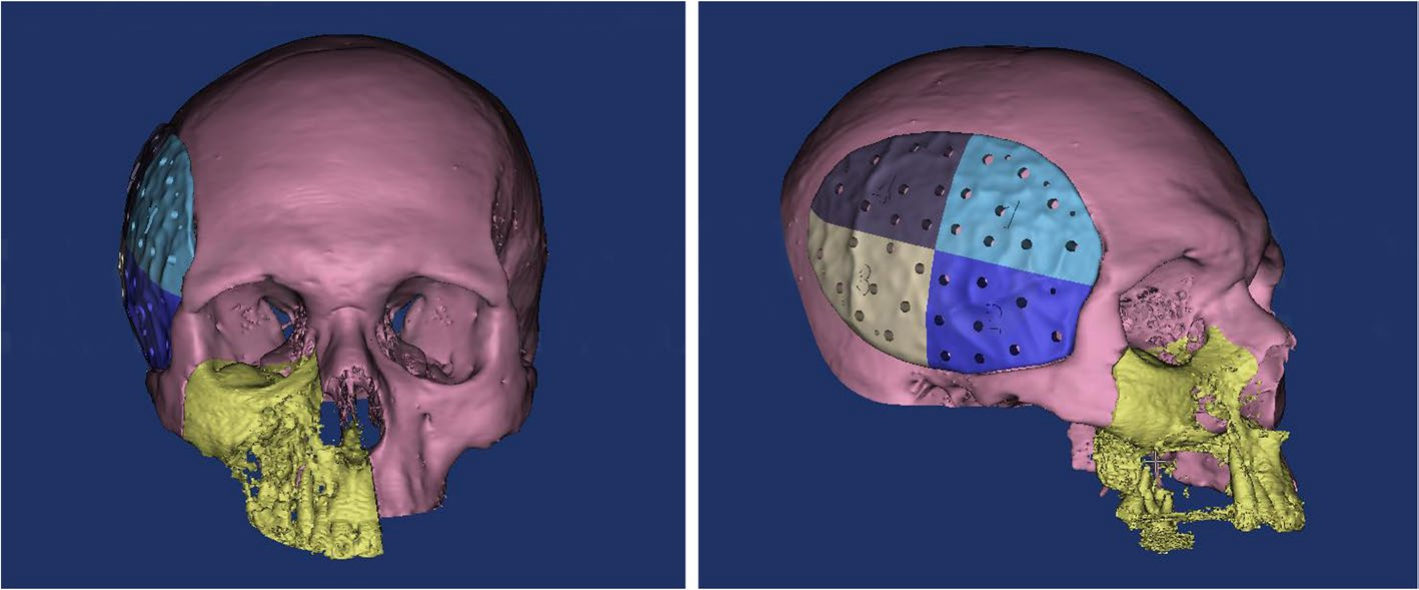
The virtual implant formed of four interlocking parts

**Fig. 4:**
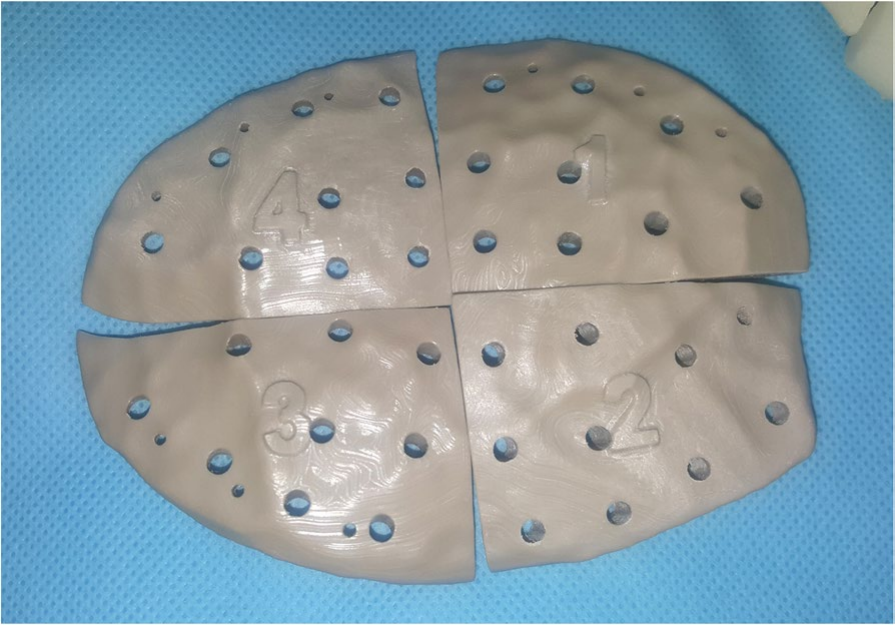
The printed patient-specific PEEK implants

Preoperative computed tomography (CT) was performed for preoperative assessment and virtual planning. CT was performed for the skull using a multi-slice helical CT machine. Image acquisition was 1 mm slice thickness, 0.625 mm slice increment, 0.3 mm voxel size, 17 × 23 cm extended field of view. DICOM files were imported to the 3D surgical planning software (Mimics 19.0, Materialise NV, Leuven, Belgium) and 3-Matic (Materialise 3-matic virtual software, Materialise NV, Leuven, Belgium) for virtual planning. Using Mimics software, the skull and the temporalis muscle were virtually segmented and separated through a series of segmentation and simulation processes (Fig. 1). The virtual muscle was imported to the 3-Matic software, and using “smoothening,” “triangular reduction,” and “adaptive remesh” tools the muscle was refined to create the final virtual temporal implant (Fig. 2). Each temporal implant was designed as four interlocking parts to accommodate for the PEEK blocks size (Fig. 3). Virtual temporal implant (STL) files were exported to a five-axis milling machine and fabricated from PEEK blocks (Fig. 4).

**Fig. 5:**
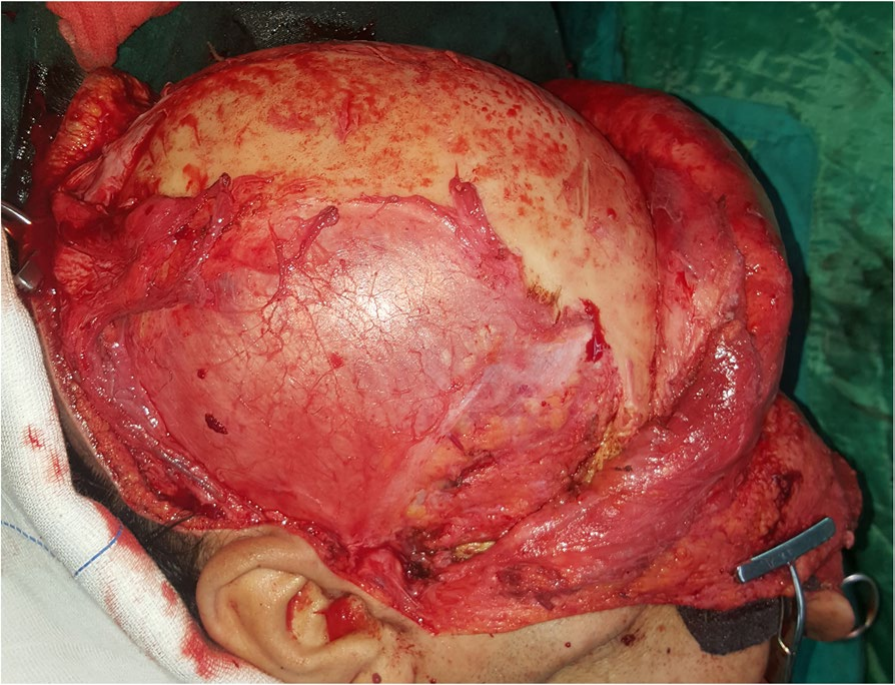
The temporalis muscle before elevation

**Fig. 6:**
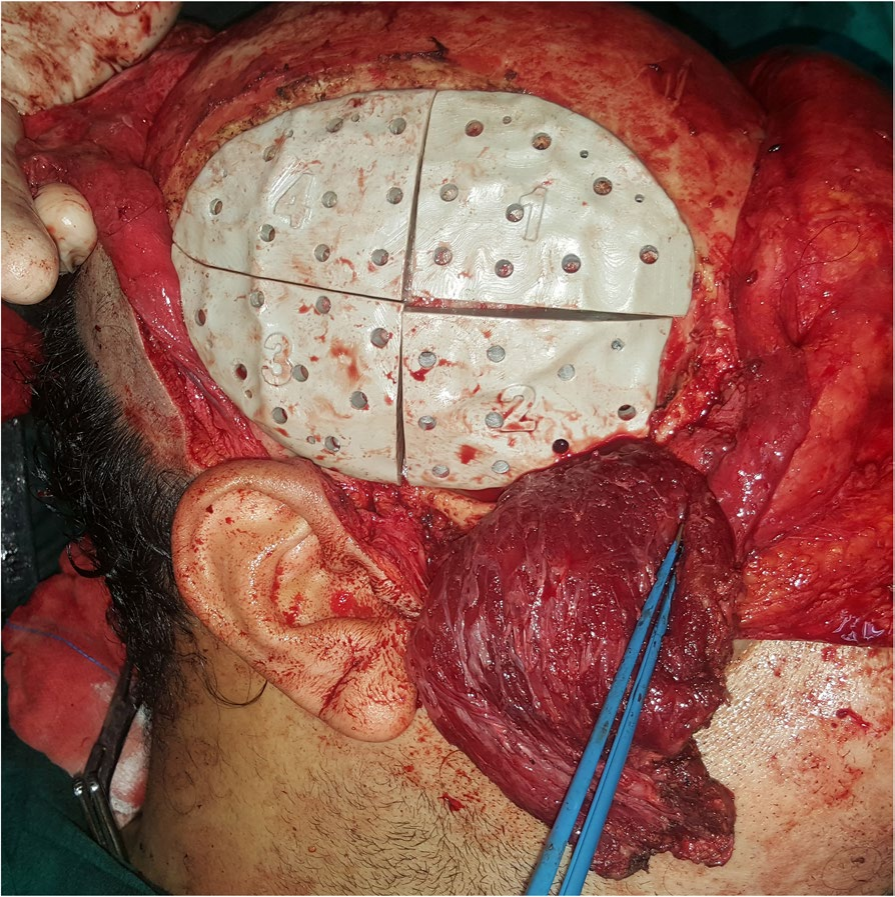
The temporalis muscle after elevation and the PEEK implant parts initially seated in place

**Fig. 7:**
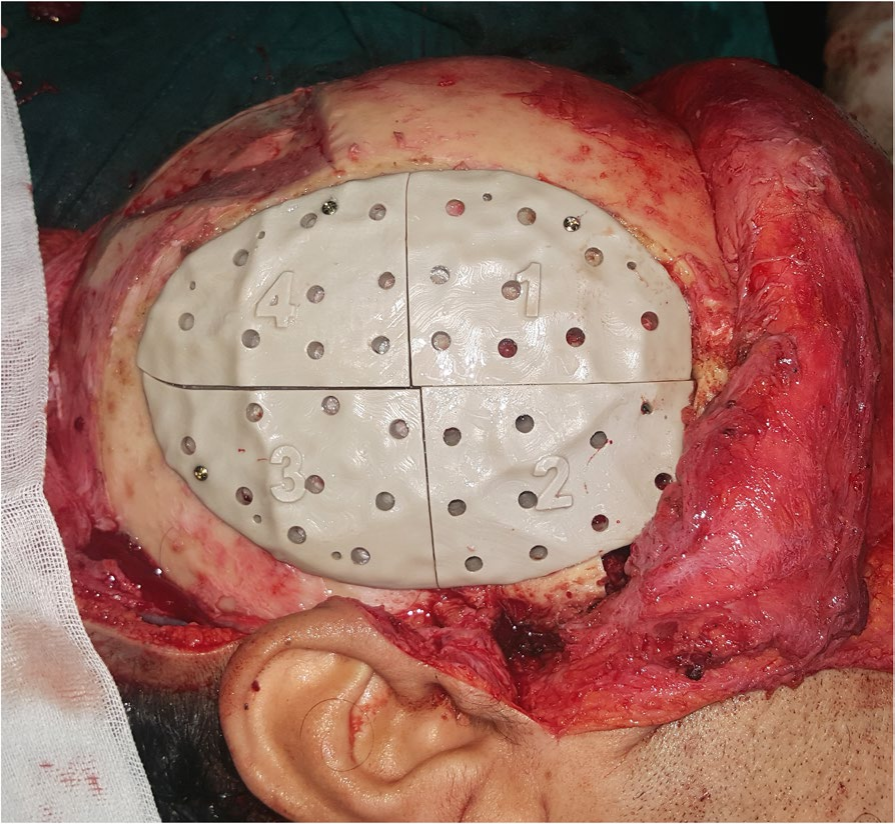
The PEEK implant fixed in position

**Fig. 8:**
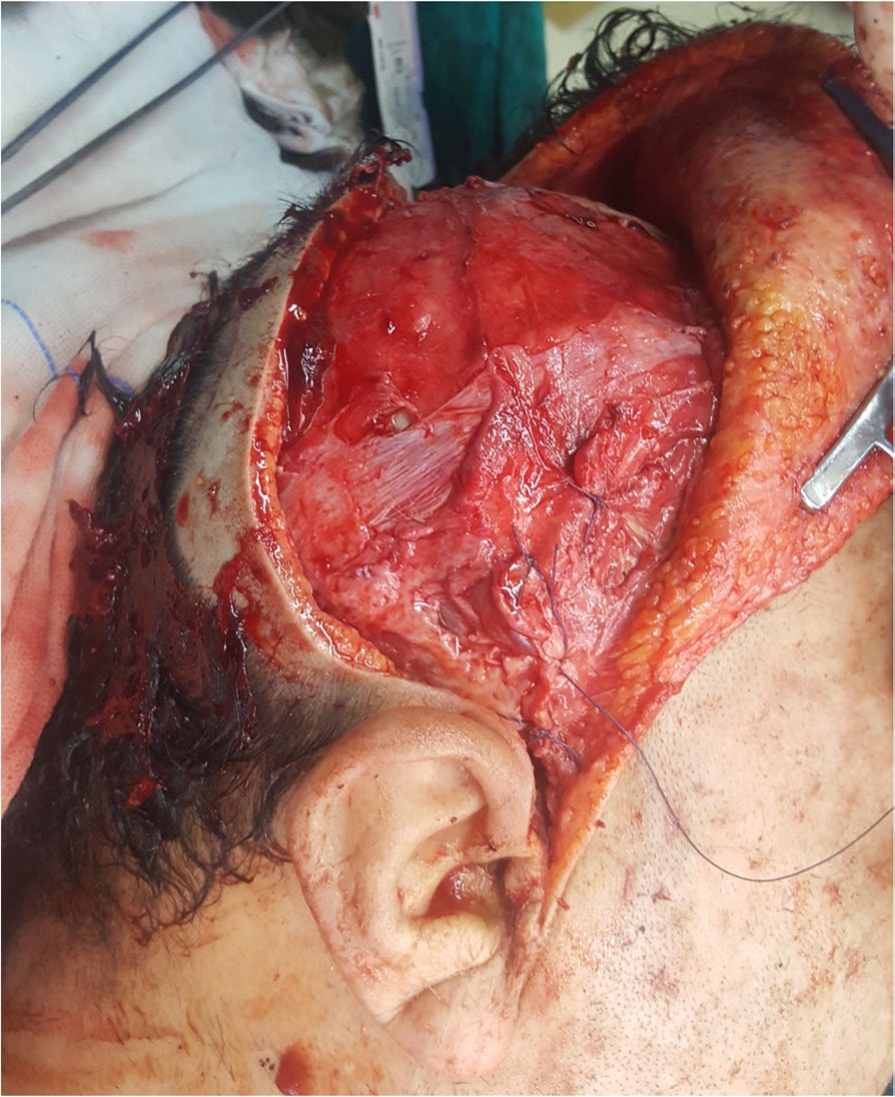
Incisions sutured in layers

Surgical procedures were performed under general anesthesia. A bi-coronal incision was marked with an inferior extension in the preauricular areas. Dissection was carried out in a superior-inferior direction in a subgaleal plane. The preauricular incision was extended down to the zygomatic arch in the subperiosteal plane preserving the facial nerve. A horizontal incision was made below the temporal crest through the temporalis fascia to expose the temporalis muscle. Blunt dissection was done to separate the temporalis fascia from its muscle, reaching below the zygomatic arch (Fig. 5). The zygomatic arch was then osteotomized to allow flap rotation into the ipsilateral maxillary defect. After flap elevation, a tunnel was created to deliver the flap to the desired location. The flap was then delivered and sutured in place. The four parts of the patient-specific PEEK temporal implant were seated in their position and fixed with screws (Figs. 6 and 7). The zygomatic arch bony segment was then repositioned and fixed with a titanium plate. Finally, the temporalis fascia was suspended in the pericranial flap and the PEEK implant, and incisions were sutured in layers (Fig. 8).

**Fig. 9:**
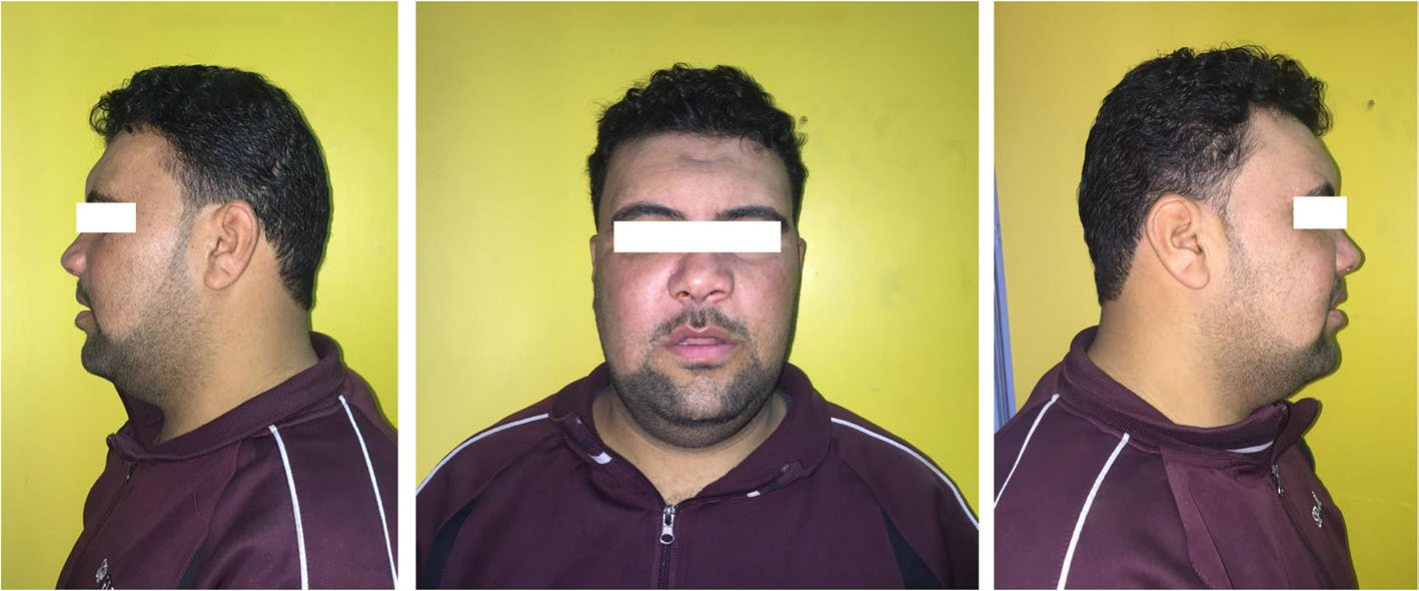
Photograph of the patient 4 weeks after the surgery

Postoperative antibiotics, steroids, and analgesics were administered for one week. All patients were followed up clinically for at least 18 months to record any postoperative complications (Fig. 9). Patient and surgeon satisfaction was assessed 4 weeks after the surgery. A five points Likert scale (1: very unsatisfied, 2: unsatisfied, 3: neutral, 4: satisfied 5: very satisfied) was used to assess the patient’s satisfaction with the implant contour and facial esthetics. Another assessment was performed by a surgeon who was not involved in the surgical procedures to detect any asymmetry and the patients were classified into three grades (I, no hollowing; II, mild hollowing; III, severe hollowing).

## Results

This study was conducted on eight patients (three females and five males) with a mean age of 45.5 ± 11.2 years. In four patients the temporalis muscle flap was used for immediate reconstruction with the maxillary defect, while in the other four patients the temporalis muscle flap was used for secondary reconstruction of the existing maxillary defect. The surgical procedures were uneventful for all patients. Post-operative clinical follow up was uneventful for all patients except one patient who showed seroma. Seroma developed after 2 weeks and resolved after serial aspirations. Patients were satisfied by the esthetic results of the temporal implants and were graded by the assessing surgeon as grade I with no temporal hollowing (Table 1).

**Table 1:** Baseline data and outcomes for individual cases

Case	Age/sex	Defect		Complications	Patient satisfaction	Surgeon grading
		Diagnosis	Reconstruction			
1	36/M	Central giant cell granuloma	Primary	-	5	I
2	62/M	Mucormycosis	Secondary	-	5	I
3	40/M	Central giant cell granuloma	Primary	-	5	I
4	53/F	Pleomorphic adenoma	Primary	-	5	I
5	30/M	Gun-shoot injury	Secondary	Seroma	4	I
6	57/F	Osteomyelitis	Secondary	-	5	I
7	48/M	Ameloblastoma	Primary	-	4	I
8	38/F	Schwannoma	Secondary	-	5	I

## Discussion

The temporal fossa is surrounded by the skull temporal ridge, the lateral orbital rim, and the zygomatic arch, and is mainly occupied by the temporalis muscle [6]. Consequently, temporalis muscle transfer for reconstructing different maxillofacial defects can result in temporal hollowing, especially when the whole muscle is used [3]. In this study, we used computer-assisted technology to fabricate patient-specific PEEK implants for immediate restoration of temporal fossa after maxillary reconstruction with temporalis muscle flap aiming to facilitate the reconstruction procedure.

Different autogenous and biomaterials have been previously used to reconstruct the temporal area either secondary to augment an exciting deformity and restore the normal contour, or primarily to prevent temporal hollowing and immediately reconstruct the temporalis muscle donor site [7, 10, 12–14].

Calvarial onlay grafts have been used to prevent temporal hollowing during pterional craniotomy [14, 15]. The major limitation of this approach was the difficulty in determining the desired graft size, and graft handling during the augmentation procedure [12]. Moreover, this approach is considered extensive in cases where the temporal augmentation is indicated for esthetic reasons after temporalis muscle transfer with no craniotomy. Autologous fat harvested from the abdominal wall has been used by Cervelli et al. [10] to correct temporal hollowing secondary to temporalis muscle transfer. The result was initially satisfactory; however, a second procedure was indicated for 78 % of the patients, and a third procedure was performed for one patient. Moreover, this approach is applicable only to correct existing temporal hollowing, not to prevent it during the temporalis muscle flap transfer [10].

Polymethyl methacrylate (PMMA) and Porous high-density polyethylene (PHDPE) are considered as the most used biomaterial for temporal hollowing correction [6, 7, 13, 16–20]. PMMA has been used for orthopedic reconstruction in 1950s. It showed initial questionable clinical results, this urged the manufacturers to improve its biological and mechanical properties. Since then, bone cement has become widely used for orthopedic prostheses [21, 22]. However, PMMA showed satisfactory esthetic results in temporal hollowing reconstruction, it showed more postoperative complications compared to PHDPE implants [3]. PHDPE was developed in the 1970s, became available for clinical implantation since the 1990s. Since then, it has been used for the augmentation and reconstruction of different craniomaxillofacial regions [23, 24]. Aside from PMMA and PHDPE, limited cases are available on other biomaterials for temporal fossa reconstruction such as porcine collagen matrix (Permacol), Mersilene mesh, and titanium implant [8, 9, 14].

In our study, we used PEEK to fabricate the temporal implants. PEEK was first developed in 1978 and used as aircraft and turbine blades. Later in the 1990s, it was used to replace metal implants [25]. It showed to be a strong and thermoplastic material. Its elasticity and energy-absorbing properties are closer to the bone compared to titanium [26]. Nowadays, PEEK is considered the gold standard for patient-specific implants. It showed promising results for the reconstruction of different craniomaxillofacial defects [23, 27–31]. However, its major limitation of the high cost of the compared to PMMA and PHDPE [23].

Different methods have been used for temporal implants fabrication and adaptation. For PHDPE temporal implants (Medpor implants), the first step was to select an implant of appropriate size. The implant was then carefully carved to the required shape, feathered to ensure that its border is not visible or palpable, and finally placed to fill the fossa [7, 22–24]. While, PMMA implants are usually directly molded using cold cure acrylic cement into the required shape and placed over the bare temporal bone after temporalis muscle transfer [6, 13, 17]. Falconer and Phillip [16] in their study used a prefabricated acrylic prothesis. A wax template for the prothesis was made on a dry skull of average proportions, then the wax was processed in heat-cured acrylic to fabricate the acrylic prothesis. Laloze et al. [14] in a case report used a preliminary PMMA spacer to shape a Permacol plate which was used for reconstruction. Hatamleh et al. [8] in another case report used a 3D model of the patient skull to shape a titanium sheet for temporalis contour reconstruction.

In our study, we used patient-specific PEEK temporal implants to immediately restore the temporal fossa contour. Prefabricated patient-specific implants have been proved to reduce the operation time and produce excellent cosmetic results [32]. We used CAS to mimic the temporalis muscle before its transfer and fabricate the implant. The muscle was virtually selected, separated, and refined to construct the virtual implant. Each implant was formed of four parts to accommodate for the PEEK blocks size. The surgical procedure was uneventful and the esthetic result was satisfactory. Different post-operative complications have been reported after temporal reconstruction as seroma, infection, temporal depression, dehiscence, and implant removal [3]. In our study, post-operative clinical follow up was uneventful for all patients except one patient who showed seroma which resolved with serial aspirations. Seroma is the collection of exudative fluid below the flap in large-detachment surgeries. The detachment of large tissue during flap elevation and residual dead space are contributory factors for its formation. Seroma is not a serious of complication but if not drained may evolve to wound dehiscence, implant extrusion, infection, and finally loss of reconstruction [33].

Our approach seems to avoid adverse events associated with intraoperative molding of PMMA [32]. Moreover, it facilitates the surgical procedures when compared to PHDPE implants. Patient-specific implants eliminate several meticulous steps that are mainly based on the surgeon’s experience as implant selection, adaptation, trimming, and feathering [10, 18, 19]. However, the major limitation of this approach is the relatively high cost of the patient-specific PEEK implants.

Within the limitations of this study, patient-specific PEEK implants represent a promising method for immediate restoration of the temporal fossa after temporalis muscle transfer. However, we recommend the conduction of more investigations and comparative studies for further evaluation of its benefits compared to other implants.

## Data Availability

The dataset supporting the conclusions of this article is available.

## References

[1] Lam D, Carlson ER (2014). The temporalis muscle flap and temporoparietal fascial flap. Oral Maxillofac Surg Clin North Am.

[2] Edwards SP, Feinberg SE (2003). The temporalis muscle flap in contemporary oral and maxillofacial surgery. Oral Maxillofac Surg Clin North Am.

[3] Laloze J, Brie J, Chaput B, Usseglio J (2019). Depression after temporal muscle flap: a systematic review of the literature. J Craniomaxillofac Surg.

[4] Habel G, Hensher R (1986). The versatility of the temporalis muscle flap in reconstructive surgery. Br J Oral Maxillofac Surg.

[5] Abubaker AO, Abouzgia MB (2002). The temporalis muscle flap in reconstruction of intraoral defects: an appraisal of the technique. Oral Surg Oral Med Oral Pathol Oral Radiol Endod.

[6] Cheung LK, Samman N, Tideman H (1994). The use of mouldable acrylic for restoration of the temporalis flap donor site. J Craniomaxillofac Surg.

[7] Rapidis AD, Day TA (2006). The use of temporal polyethylene implant after temporalis myofascial flap transposition: clinical and radiographic results from its use in 21 patients. J Oral Maxillofac Surg.

[8] Muhanad H, Jason W, Andrew R, Dilip S (2013). A novel approach to immediate restoration of the cosmetic deformity after regional temporalis flap reconstruction of a maxillary defect: a case report. J Craniofac Surg.

[9] Donald AD, Naresh J, Niall K (2010). Augmentation of temporal fossa hollowing with mersilene mesh. J Plast Reconstr Aesthet Surg.

[10] Cervelli D, Gasparini G, Grussu F, Moro A, Marianetti TM, Foresta E, Azzuni C, Pelo S (2014). Autologous fat transplantation for the temporalis muscle flap donor site: our experience with 45 cases. Head Neck.

[11] Ahmed M, Soliman S, Noman SA, Ali S (2020). Computer-guided contouring of craniofacial fibrous dysplasia involving the zygoma using a patient-specific surgical depth guide. Int J Oral Maxillofac Surg.

[12] Kim JH, Lee R, Shin CH, Kim HK, Han YS (2018). Temporal augmentation with calvarial onlay graft during pterional craniotomy for prevention of temporal hollowing. Arch Craniofac Surg.

[13] Mandlik D, Gupta K, Patel D, Patel P, Toprani R, Patel K (2015). Use of Polymethyl Methacrylate-Based Cement for Cosmetic Correction of Donor-Site Defect following Transposition of Temporalis Myofascial Flap and Evaluation of Results after Adjuvant Radiotherapy. J Reconstr Microsurg.

[14] Laloze J, Brie J, Chaput B, Usseglio J (2020). Use of PermacolTM to restore depression after temporal muscle flap: A case report. J Stomatol Oral Maxillofac Surg.

[15] Kim YS, Yi HS, Kim HK, Han YS (2019). Effectiveness of Temporal Augmentation Using a Calvarial Onlay Graft during Pterional Craniotomy. Arch Plast Surg.

[16] Falconer DT, Phillips JG (1991). Reconstruction of the defect at the donor site of the temporalis muscle flap. Br J Oral Maxillofac Surg.

[17] Wright S, Bekiroglu F, Whear NM, Grew NR (2006). Use of Palacos®R-40 with gentamicin to reconstruct temporal defects after maxillofacial reconstructions with temporalis flaps. Br J Oral Maxillofac Surg.

[18] Lacey M, Antonyshyn O (1993). Use of porous high-density polyethylene implants in temporal contour reconstruction. J Craniofac Surg.

[19] Worley CM, Strauss RA (1994). Augmentation of the anterior temporal fossa after temporalis muscle transfer. Oral Surg Oral Med Oral Pathol.

[20] Baj A, Spotti S, Marelli S, Beltramini GA, Giannì AB (2009). Use of porous polyethylene for correcting defects of temporal region following transposition of temporalis myofascial flap. Acta Otorhinolaryngol Ital.

[21] Vaishya R, Chauhan M, Vaish A (2013). Bone cement. J Clin Orthop. Trauma.

[22] Arora M, Chan EK, Gupta S, Diwan AD (2013). Polymethylmethacrylate bone cements and additives: A review of the literature. World J Orthop.

[23] Ridwan-Pramana A, Wolff J, Raziei A, Ashton-James CE, Forouzanfar T (2015). Porous polyethylene implants in facial reconstruction: Outcome and complications. J Craniomaxillofac Surg.

[24] Mansour Khorasani M, Janbaz P, Rayati F (2018). Maxillofacial reconstruction with Medpor porous polyethylene implant: a case series study. J Korean Assoc Oral Maxillofac Surg.

[25] Honigmann P, Sharma N, Okolo B, Popp U, Msallem B, Thieringer FM (2018). Patient-Specific Surgical Implants Made of 3D Printed PEEK: Material, Technology, and Scope of Surgical Application. Biomed Res Int.

[26] Jonkergouw J, Van de Vijfeijken SECM, Nout E, Theys T, Van de Casteele E, Folkersma H, Depauw PRAM, Becking AG (2016). Outcome in patient-specific PEEK cranioplasty: A two-center cohort study of 40 implants. J Craniomaxillofac Surg.

[27] Punchak M, Chung LK, Lagman C, Bui TT, Lazareff J, Rezzadeh K, Jarrahy R, Yang I (2017). Outcomes following polyetheretherketone (PEEK) cranioplasty: Systematic review and meta-analysis. J Clin Neurosci.

[28] Jalbert F, Boetto S, Nadon F, Lauwers F, Schmidt E, Lopez R (2014). One-step primary reconstruction for complex craniofacial resection with PEEK custom-made implants. J Craniomaxillofac Surg.

[29] Alonso-Rodriguez E, Cebrián JL, Nieto MJ, Del Castillo JL, Hernández-Godoy J, Burgueño M (2015). Polyetheretherketone custom-made implants for craniofacial defects: Report of 14 cases and review of the literature. J Craniomaxillofac Surg.

[30] Gerbino G, Zavattero E, Zenga F, Bianchi FA, Garzino-Demo P, Berrone S (2015). Primary and secondary reconstruction of complex craniofacial defects using polyetheretherketone custom-made implants. J Craniomaxillofac Surg.

[31] Järvinen S, Suojanen J, Kormi E, Wilkman T, Kiukkonen A, Leikola J, Stoor P (2019). The use of patient specific polyetheretherketone implants for reconstruction of maxillofacial deformities. J Craniomaxillofac Surg.

[32] Dodier P, Winter F, Auzinger T, Mistelbauer G, Frischer JM, Wang WT, Mallouhi A, Marik W, Wolfsberger S, Reissig L, Hammadi F, Matula C, Baumann A, Bavinzski G (2020). Single-stage bone resection and cranioplastic reconstruction: comparison of a novel software-derived PEEK workflow with the standard reconstructive method. Int J Oral Maxillofac Surg.

[33] Maricevich JPBR, Cezar AB, De Oliveira EX, Silva JAMV, Maricevich RS, Almeida NS, Azevedo-Filho HRC (2018). Adhesion sutures for seroma reduction in cranial reconstructions with polymethyl methacrylate prosthesis in patients undergoing decompressive craniectomy: A clinical trial. Surg Neurol Int..

